# T2 mapping outperforms normalised FLAIR in identifying hippocampal sclerosis

**DOI:** 10.1016/j.nicl.2015.03.004

**Published:** 2015-03-13

**Authors:** R. Rodionov, P.A. Bartlett, Ci He, S.B. Vos, N.K. Focke, S.G. Ourselin, J.S. Duncan

**Affiliations:** a Department of Clinical and Experimental Epilepsy, UCL Institute of Neurology, London, UK; b MRI Unit, Epilepsy Society, Chalfont St Peter, Buckinghamshire, UK; c Department of Radiology, Chengdu Military General Hospital, China; d Centre for Medical Image Computing, Translational Imaging Group, University College London, London, UK; e Department of Neurology and Epileptology, Hertie-Institute for Clinical Brain Research, University of Tübingen, Tübingen, Germany

**Keywords:** Hippocampal sclerosis, T2 FLAIR, T2 mapping

## Abstract

**Rationale:**

Qualitatively, FLAIR MR imaging is sensitive to the detection of hippocampal sclerosis (HS). Quantitative analysis of T2 maps provides a useful objective measure and increased sensitivity over visual inspection of T2-weighted scans. We aimed to determine whether quantification of normalised FLAIR is as sensitive as T2 mapping in detection of HS.

**Method:**

Dual echo T2 and FLAIR MR images were retrospectively analysed in 27 patients with histologically confirmed HS and increased T2 signal in ipsilateral hippocampus and 14 healthy controls. Regions of interest were manually segmented in all hippocampi aiming to avoid inclusion of CSF. Hippocampal T2 values and measures of normalised FLAIR Signal Intensity (nFSI) were compared in healthy and sclerotic hippocampi.

**Results:**

HS was identified on T2 values with 100% sensitivity and 100% specificity. HS was identified on nFSI measures with 60% sensitivity and 93% specificity.

**Conclusion:**

T2 mapping is superior to nFSI for identification of HS.

## Highlights

• T2 mapping has significantly outperforms normalised FLAIR in differentiating sclerotic from healthy hippocampi.
• Due to high sensitivity and specificity T2 mapping carries high potential to point out potential bilateral pathology.
• The difference of ability to detect the pathology by the two techniques is likely to hold for advanced FLAIR sequences.
• T2 mapping should be the technique of choice to differentiate hippocampal sclerosis in clinical setting.

## 1 Introduction

Increased signal intensity on T2 weighted MR images is one of the diagnostic criteria for hippocampal sclerosis (HS) (Cendes, 2013). FLAIR MR imaging is a popular and sensitive method for identifying cerebral parenchymal abnormalities, including HS (Duncan, 2010).

Quantification of hippocampal T2 signal is useful as an objective measure of hippocampal pathology (Woermann et al., 1998) that is useful for research purposes and clinically, both to identify subtle abnormalities and to determine if the contralateral hippocampus is normal when hippocampal resection is planned for treatment of epilepsy.

Hippocampal T2 mapping has been used to good effect for many years, and can be estimated using a dual echo sequence (Bartlett et al., 2007). It would be advantageous if quantification of FLAIR signal would achieve these aims, as the sequence is routinely acquired clinically. Mapping of normalised FLAIR signal intensity (nFSI) has been shown to be sensitive to detection of malformations of cortical development and also assists the detection of subtle cortical lesions (Focke et al., 2008, 2009). In one study, mapping hippocampal FLAIR signal intensity (Huppertz et al., 2011) showed good separation between patients with HS and healthy controls.

We aimed to check if normalised FLAIR technique is as sensitive and specific as clinically validated T2 mapping technique in the task of the detection of HS.

## 2 Method

### 2.1 Subjects

We analysed MRI data from three cohorts of subjects, comprising patients with histologically confirmed right (13) HS, left (14) HS and elevated T2 values measured in vivo with a dual echo method (Bartlett et al., 2007), and 14 healthy controls. The median (range) of age of the controls was 44 (24–61) years. The histological analysis of hippocampal tissue removed during the resective surgery confirmed HS in all patients with two left HS patients having triple and dual pathology (1 — HS, focal cortical dysplasia, ganglioglioma; 2 — HS, dysembryoplastic neuroepithelial tumour). The characteristics of the patient group are summarised in Table 1.

The patients were scanned for clinical purposes using the established clinical protocol that included T2 FLAIR and dual T2 images. The Research Ethics Committee did not require individual patient consent as the study was classified as a service evaluation. The HS was diagnosed preoperatively on MRI of all patients and confirmed histologically. Healthy controls gave written, informed consent.

### 2.2 Imaging

The images utilised for the analysis were acquired on a GE Excite 3T MRI scanner with an 8-channel head coil.

T2 Fluid Attenuated Inversion Recovery (FLAIR): Fast Spin Echo (FSE), TE 140 ms, TR 11,000 ms, TI 2250, NEX = 1, FOV 24 × 24 cm, 32 slices, 256 × 256 matrix size, voxel size 0.94 × 0.94 × 5 mm, and scan duration 2 min 57 s.

Dual-echo T2 mapping: Fast Relaxation Fast Spin Echo (FRFSE), TR 2000 ms, TE 30 and 80 ms, FOV 24 × 24 cm, 32 slices, 256 × 256 matrix size; voxel size 0.94 × 0.94 × 5 mm, and scan duration 3 min 1 s.

**Fig. 1:**
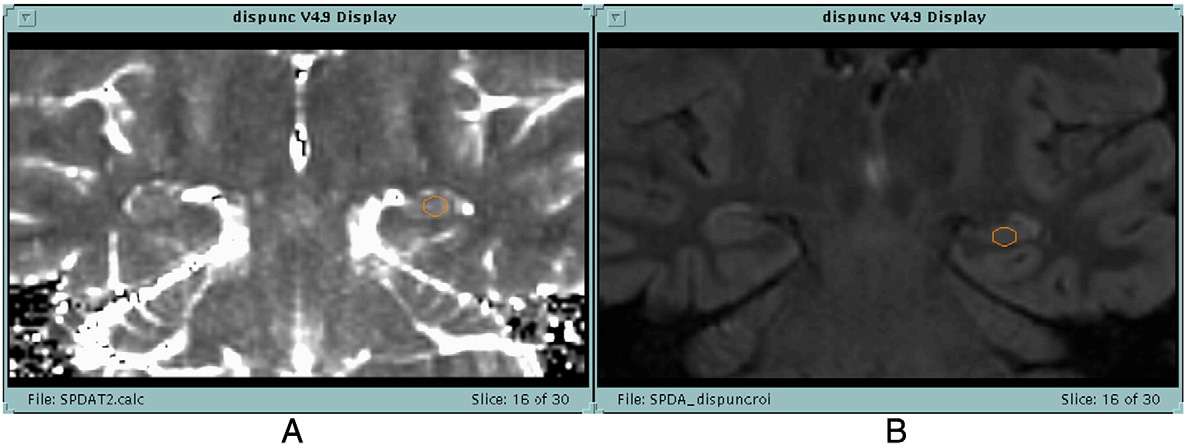
Placement of ovoid ROIs on hippocampal slices of T2 map A and T2 FLAIR (B) images (Bartlett et al., 2007).

Both scans were oriented in the oblique coronal plane in the same axis as the brain stem, orthogonal to the hippocampus, so as to minimise partial volume effects (Fig. 1).

### 2.3 Data analysis

Pixel-by-pixel T2 maps were calculated from the FRFSE images by using the expression T2 = (TE2 − TE1) / [ln(S1 / S2)], where S1 and S2 are the signal intensity in the early- and late-echo images, with echo times TE1 = 30 ms and TE2 = 80 ms, respectively (Bartlett et al., 2007).

An ovoid hippocampal region of interest (ROI) was placed manually within the hippocampus both on FLAIR and T2 map images, in 4–5 consecutive 5 mm thick coronal slices from posterior to anterior. The ROI was at least 18 mm^2^ and as large as could be accommodated within the outline of the hippocampus while avoiding CSF and partial volume effect with CSF. The T2 map and T2 FLAIR images were arranged on the screen next to each other in the process of segmentation (Fig. 1), to ensure that the ROIs were placed in the same parts of the two series of scans.

The average signal intensities were measured across each coronal slice of each hippocampal ROI of both T2 FLAIR and T2 images. The data from each individual slice were averaged to yield a single measure for each hippocampus.

The hippocampal ROI measures obtained from the T2 FLAIR were normalised using the average T2 FLAIR signal intensity across white matter in pons and anterior frontal lobes as described previously for nFSI analysis (Focke et al., 2008, 2009). The nFSI image processing was implemented using FSL (FMRIB, Oxford, UK).

## 3 Results

**Fig. 2:**
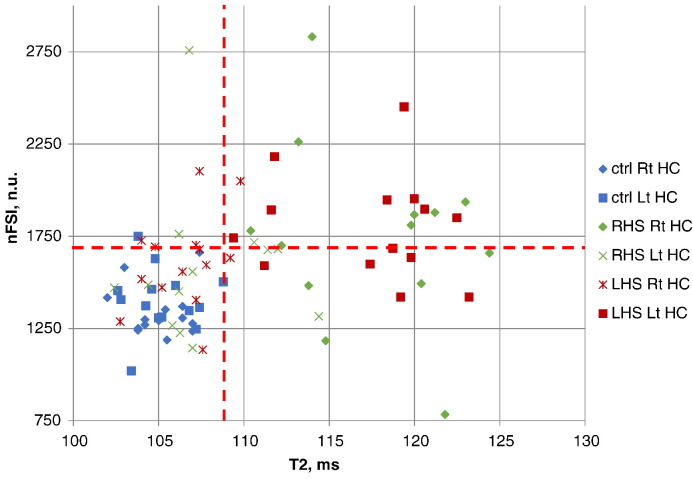
Comparison of T2 and nFSI values for separation of healthy-vs-sclerotic hippocampi. Horizontal axis — T2 values; vertical axis — nFSI values. The red dashed lines, defined as mean + 2SD, separate measurements for the hippocampi of the healthy controls.

Fig. 2 illustrates the comparison of separation between normal (controls; 28) and diseased (histologically confirmed HS in patients; 27) hippocampi based on T2 (horizontal axis) and nFSI (vertical axis) values. The upper limit of normal hippocampal T2 in this cohort, defined as mean + 2SDs, was 109 ms with one normal hippocampus on the borderline (108.8 ms). Sclerotic hippocampi had T2 values ranging from 109.4 to 124.4 ms with one of them very close to the normal limit.

The upper limit of normal of nFSI, defined as mean + 2SDs, was 1683 normalised units. The nFSI of one control has reached 1750 n.u. exceeding the normal limit. 11/27 sclerotic hippocampi had nFSI values below the normal limit with one very close to the borderline (1680 n.u.).

**Fig. 3:**
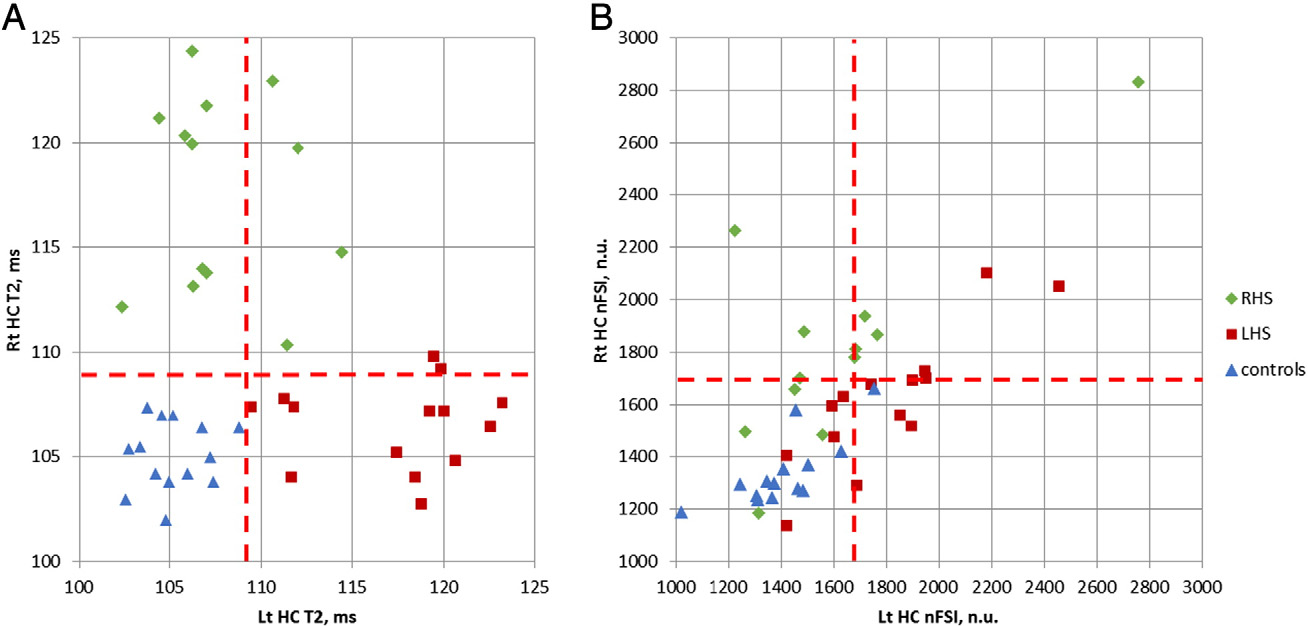
The comparison of T2 (A) and nFSI (B) values for lateralisation of the HS and separation controls-vs-patients. The red dashed lines, defined as mean + 2SD, separate measurements for the hippocampi of the healthy controls.

Fig. 3 demonstrates the ability of lateralisation of HS and separation between healthy controls and patients for each imaging technique individually (a — T2 mapping; b — nFSI). Note the possibility to draw the separating limits on a T2 values (Fig. 3a) plot in a way which allows avoiding both false positives and negatives.

The healthy hippocampi were differentiated from sclerotic hippocampi proved histologically based on:

T2 values: 100% sensitivity and 100% specificity;
nFSI values: 60% sensitivity and 93% specificity,

where specificity and sensitivity were calculated based on the thresholds shown in Fig. 3 (Campbell et al., 2007).

As expected, in some patients the hippocampus contralateral to HS had abnormal T2 values in 6/27 (22%) cases (Fig. 2), reflecting the fact that HS may be bilateral.

## 4 Discussion

We compared the sensitivity and specificity of normalised FLAIR and T2 mapping techniques in the task of the detection of HS based on the cohort of patients with histologically confirmed HS and elevated T2 signal in the ipsilateral hippocampus.

The results demonstrate a higher rate of differentiation between healthy and sclerotic hippocampi when using dual-echo T2 mapping technique compared to nFSI.

We applied the same method of analysis for images of both sequences in order to concentrate on comparison of the imaging techniques rather than the specificities of the analysis. The only difference — the step of normalisation of the T2 FLAIR image — was unavoidable as the FLAIR image is not quantitative.

The accurate segmentation of hippocampal ROI is crucial for this study. Despite the recent significant advances in automated segmentation of hippocampi (Winston et al., 2013) the manual segmentation of sclerotic hippocampi with emphasis on morphology remains the gold standard. Manual placement of an ovoid hippocampal ROI allowed avoiding inclusion of high T2 signal from CSF around hippocampus. The choroid plexus may give rise to false positive high signal when erroneously segmenting posterior section of hippocampus.

Therefore, our choice of the analysis algorithm was predefined by the method of manual segmentation and the technique which has been successfully used in clinical practice (Bartlett et al., 2007).

The two cases with additional pathology (1 — HS, focal cortical dysplasia, ganglioglioma; 2 — HS, dysembryoplastic neuroepithelial tumour) were the only left HS cases showing weak evidence for bilateral HS according to the T2 mapping results (the 2 red labels above the horizontal threshold line on the Fig. 3a). The separation from controls based on nFSI was successful in one of these two cases. These results do not show difference from the results observed in cases of single pathology when the measurements taken from the resected hippocampi are considered.

The different nFSI algorithm has been successfully applied for the differentiation of controls from patients with HS (Huppertz et al., 2011). The authors reported 97% sensitivity with 95% specificity for the differentiation of controls from patients with 95% confidence. The significant difference from our result can be related to the differences in the analysis algorithms of which the key are the definition of ROI, FLAIR image normalisation, approach to set up statistical limits between controls and patients. The ROI in our study are significantly smaller due to the necessity to avoid partial volume effects with CSF appearing as high signal on T2 images. In addition, any approaches based on applying masks of higher spatial resolution in the through-plane direction would potentially lead to significant signal changes around the edges of such ROI due to interpolation. It is important in this context that the slice thickness of our T2 and FLAIR images is twice that in the data from Dr. Huppertz.

Other factors contributing to the difference of the results, such as different cohorts of patients and use of a different scanner, may also contribute to this difference. It is noteworthy that both the scatterplot (Fig. 3b) and the analogues scatterplot (see Figure 2 in Huppertz, 2011) show similarity in their inability to show clear quantitative limits of hippocampal nFSI measures between controls and patients. In contrast, the T2 scatterplot illustrates existence of such quantitative limits.

The T2 map is obtained based on two measurements (dual-echo sequence) whereas T2 FLAIR is based on one measurement. This is an inherent difference of the modalities, which leads to the T2 mapping technique to have twice the amount of data as the FLAIR and thus likely also a higher SNR. The performance of the nFSI method would improve with twice the sampling. However, the doubled sampling would increase the duration of the T2 FLAIR scan making it less practical clinically and more prone to the motion artefact.

It remains to be determined whether nFSI may detect abnormalities in the small minority of cases with sclerotic hippocampi in which T2 mapping is within the normal range.

## 5 Conclusion

T2 mapping remains the technique of choice for differentiation of the patients with HS from healthy subjects when applying the criterion of T2 signal intensity.

There is a high degree of confidence that this conclusion will persist even with advances with neuroimaging equipment and introduction of diagnostic T2 images with resolution at the level of 1 mm^3^ isotropic.

## Figures and Tables

**Table 1:** Characteristics of the patient group (*n* = 27).

Characteristic	Value
Age at scanning, years (median (range))	32 (18–52)
Age at onset of epilepsy, year (median (range))	11 (1–32)
Duration of epilepsy at the time of scan, year (median (range))	19 (6–46)
No of complex partial seizures per month in year prior to scan (median (range))	8 (1–900)
No of patients with habitual secondarily generalised tonic clonic seizures	13
No of patients with early childhood convulsion	13
